# Wide Complex Arrhythmia—A Unique Presentation of an Unusual Syndrome

**DOI:** 10.19102/icrm.2023.14036

**Published:** 2023-03-15

**Authors:** Rohan Trivedi, Angela Naperkowski, Sarah Worsnick, Wilson Young, Pugazhendhi Vijayaraman

**Affiliations:** ^1^Geisinger Wyoming Valley Hospital, Wilkes Barre, PA, USA

**Keywords:** Genetic testing, syncope, wide complex tachycardia

## Abstract

The mechanisms of wide complex tachycardia can vary. We discuss the case of a wide complex tachycardia with multiple mechanisms due to a rare genetic abnormality in a 26-year-old Caucasian man with a past history of spontaneous pneumothorax and syncope.

## Introduction

The mechanisms of wide complex tachycardia (WCT) can vary from ischemic, structural, electrolyte disturbance, and drug-induced to genetic, to name a few. We present a case of a WCT with multiple mechanisms due to a rare genetic abnormality.

## Case presentation

**Figure 1:**
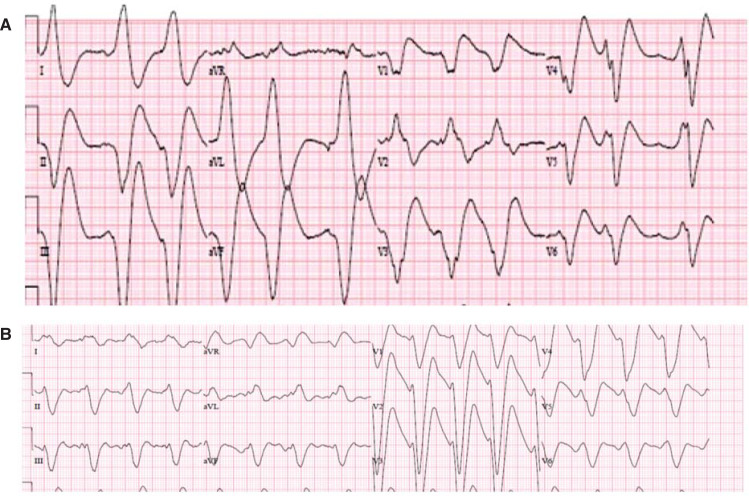
**A:** Presenting 12-lead electrocardiogram with wide complex rhythm. The heart rate is 70 bpm, with QRSd = 200 ms and QT/corrected QT = 582/639 ms. **B:** A post-cardioversion sustained wide complex arrhythmia is seen (QRSd = 400 ms).

**Figure 2:**
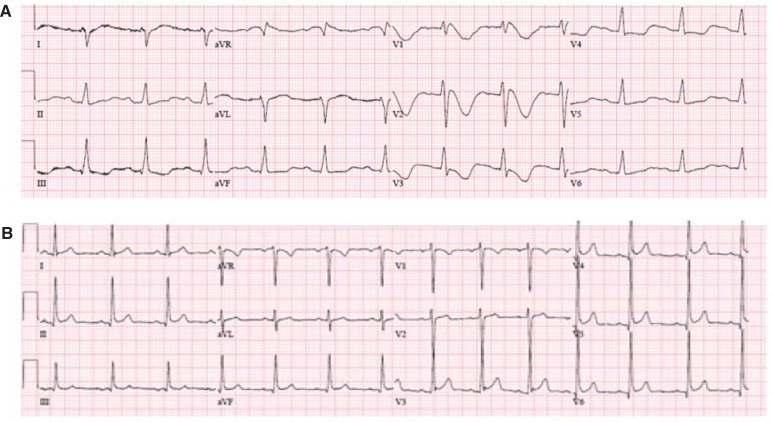
**A:** Spontaneous, transient, Brugada-like electrocardiogram. **B:** The patient’s baseline 12-lead electrocardiogram. Normal sinus rhythm with a short QT interval and early repolarization changes are noted.

We present the case of a 26-year-old Caucasian man with a past history of spontaneous pneumothorax and syncope (2 episodes, unknown etiology) who presented to the emergency department for syncope. The presenting 12-lead electrocardiogram (ECG) demonstrated a WCT **(Figure 1A)** requiring cardioversion due to hemodynamic instability. Post-conversion, he remained in sustained normal sinus with a wide QRS arrhythmia (QRS duration, 400 ms) for 12 h **(Figure 1B)**. During admission, he was observed to have several different asymptomatic rhythms, including a Brugada-like pattern **(Figure 2A)** with spontaneous resolution.

**Figure 3:**
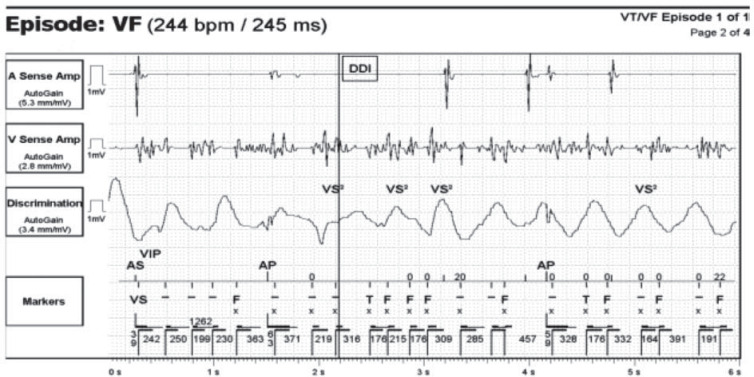
Device interrogation noting slow ventricular tachycardia and ventricular fibrillation morphology and true fractionated ventricular electrograms masquerading as right ventricular lead noise.

A baseline 12-lead ECG showed normal sinus rhythm with a short QT interval and early repolarization changes **(Figure 2B)**. The echocardiogram and stress test results were unremarkable, and cardiac magnetic resonance imaging showed no structural abnormalities or late gadolinium enhancement. The patient was started on 25 mg of metoprolol tartrate twice daily and underwent implantation of a dual-chamber implantable cardioverter-defibrillator (ICD), with no recurrent arrhythmias noted prior to hospital discharge. Three months later, he experienced recurrent WCT requiring ICD therapies. Device interrogation revealed slow ventricular tachycardia and ventricular fibrillation rhythms with severe intraventricular delayed conduction and fractionated ventricular potentials masquerading as under-sensing and right ventricular lead noise **(Figure 3)**. Subsequently, an electrophysiology study demonstrated advanced conduction system disease and prolonged QT (baseline H–V = 62 ms, QT = 540 ms @ 50 bpm).

**Figure 4:**
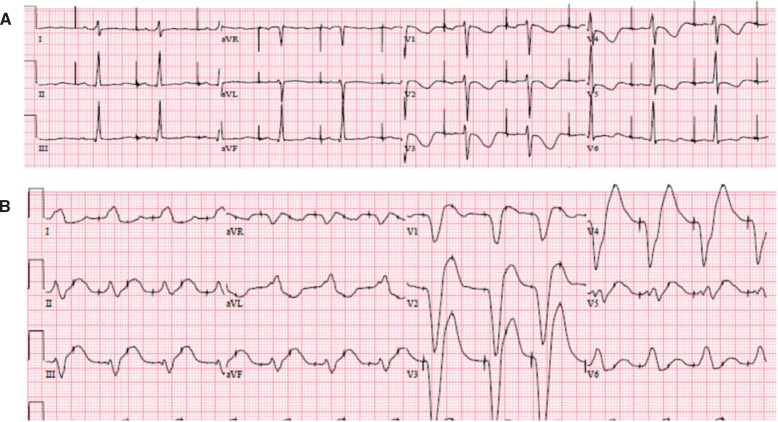
The patient displayed multiple conduction system and repolarization abnormalities; note the **(A)** sinus node dysfunction and prolonged QT interval and **(B)** under-sensing and intermittent non-capture.

**Figure 5:**
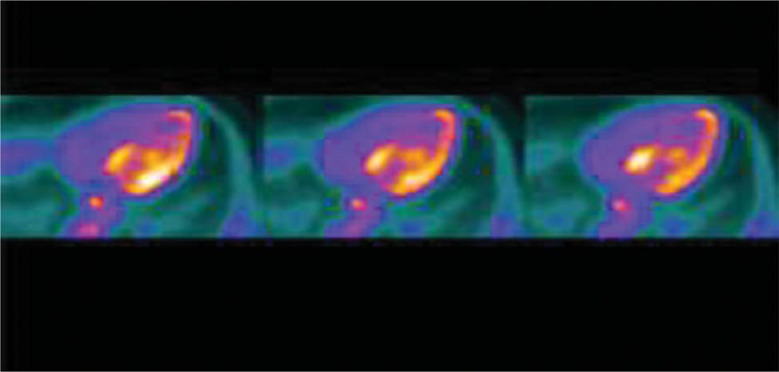
A positron emission tomography scan displaying variable patchy fluorodeoxyglucose uptake. Here, basal and apical septum and anterolateral wall uptake is visualized.

No Wolff–Parkinson–White (WPW) syndrome or reproducible arrhythmias were noted. The patient also displayed a variety of transient conduction system and myocardial conduction diseases, including sinus node dysfunction, first-degree atrioventricular (AV) block, high-grade AV block, a prolonged QT interval **(Figure 4A)**, and transient episodes of atrial and ventricular non-capture **(Figure 4B)**. The conglomeration of presenting electrical disorders garnered suspicion for possible long QT syndrome and sodium channelopathy. He was started on empiric quinidine but was unable to tolerate it due to headaches along with recurrent outpatient WCT and ICD shocks. He subsequently underwent genetic testing, which confirmed a *PRKAG2* mutation (variant of unknown significance, c131C>T—p.ALaV44Val). A positron emission tomography scan revealed patchy fluorodeoxyglucose uptake **(Figure 5)** without corresponding structural or wall motion abnormalities on the echocardiogram. He was started on a strict metabolic diet containing high protein and low carbohydrate concentrations with excellent success. He has remained arrhythmia-free for 6 years, with no further instances of WCT. He recently underwent a successful device generator change and remains asymptomatic without recurrent ICD shocks during follow-up.

## Discussion

We report a unique case of WCT secondary to *PRKAG2* mutation with successful treatment encompassing strict metabolic diet therapy. Our patient’s varied electrical abnormalities—including a long QT interval, short QT interval, a Brugada-like pattern, sinus node dysfunction, high-grade AV block, and intraventricular conduction delay with a resultant multi-morphology WCT—certainly compose an atypical presentation for this syndrome. The diagnosis of wide complex arrhythmias due to genetic etiologies remains elusive due to their low prevalence and variable presentation. Our case illuminates that, with increasing availability, genetic testing should be performed in all patients with arrhythmias of unknown etiologies and considered at earlier stages in the workup.

While our patient transiently displayed a variety of conduction system diseases, he had no evidence of left ventricular hypertrophy or a WPW pattern, which is commonly observed in *PRKAG2* syndrome.^1,2^ Furthermore, our patient presented with syncope, which was reported in 22% of *PRKAG2* patients in a prior study.^3^ Despite anti-arrhythmic drug failure, our patient’s myriad electrical disorders all proved to be reversible with a strict metabolic diet consisting of high protein and low carbohydrate levels. The *PRKAG2* mutation is a rare autosomal dominant disorder resulting in altered adenosine triphosphate metabolism, myocyte enlargement, and subsequent glycogen storage cardiomyopathy.^3^ Our case also illustrates the importance of considering an alternative diet as therapy aimed as counteracting the inherent metabolic derangements.

Our patient has remained compliant with his diet and subsequently free from arrhythmias and ICD shock therapy for 6 years.
